# Defective migration and dysmorphology of neutrophil granulocytes in atypical chronic myeloid leukemia treated with ruxolitinib

**DOI:** 10.1186/s12885-020-07130-7

**Published:** 2020-07-13

**Authors:** Lea Bornemann, Marc Schuster, Saskia Schmitz, Charlyn Sobczak, Clara Bessen, Simon F. Merz, Karl-Heinz Jöckel, Thomas Haverkamp, Matthias Gunzer, Joachim R. Göthert

**Affiliations:** 1 Institute for Experimental Immunology and Imaging, University Hospital, University Duisburg-Essen, Hufelandstrasse 55, 45147 Essen, Germany; 2 Present address: Miltenyi Biotec B.V. & Co. KG, Friedrich-Ebert-Straße 68, 51429 Bergisch Gladbach, Germany; 3 Department of Dermatology, Venerology and Allergology, University Hospital Essen, Hufelandstrasse 55, 45147 Essen, Germany; 4 Institute for Medical Informatics, Biometry and Epidemiology, University Hospital, University Duisburg-Essen, Hufelandstrasse 55, 45147 Essen, Germany; 5 MVZ Dr. Eberhard & Partner, Brauhausstraße 4, 44137 Dortmund, Germany; 6 Leibniz-Institut für Analytische Wissenschaften - ISAS -e.V, Dortmund, Germany; 7 Department of Hematology, University Hospital, West German Cancer Center (WTZ), University Duisburg-Essen, Hufelandstrasse 55, 45147 Essen, Germany

**Keywords:** aCML, Ruxolitinib, Neutrophil granulocytes, Standardized migration analysis, Cell morphology, Case report

## Abstract

**Background:**

The identification of pathologically altered neutrophil granulocyte migration patterns bears strong potential for surveillance and prognostic scoring of diseases. We recently identified a strong correlation between impaired neutrophil motility and the disease stage of myelodysplastic syndrome (MDS). Here, we apply this assay to study quantitively increased neutrophils of a patient suffering from a rare leukemia subtype, atypical chronic myeloid leukemia (aCML).

**Methods:**

A 69-year-old male was analyzed in this study. Besides routine analyses, we purified the patient’s neutrophils from peripheral whole blood and studied their migration behavior using time-lapse video microscopy in a standardized assay. These live cell migration analyses also allowed for the quantification of cell morphology. Furthermore, the cells were stained for the markers CD15, CD16, fMLPR, CXCR1 and CXCR2.

**Results:**

Despite cytoreductive therapy with hydroxyurea, the patient’s WBC and ANC were poorly controlled and severe dysgranulopoiesis with hypogranularity was observed. Neutrophils displayed strongly impaired migration when compared to healthy controls and migrating cells exhibited a more flattened-out morphology than control neutrophils. Because of a detected CSF3R (p.T618I) mutation and constitutional symptoms treatment with ruxolitinib was initiated. Within 1 week of ruxolitinib treatment, the cell shape normalized and remained indistinguishable from healthy control neutrophils. However, neutrophil migration did not improve over the course of ruxolitinib therapy but was strikingly altered shortly before a sinusitis with fever and bleeding from a gastric ulcer. Molecular work-up revealed that under ruxolitinib treatment, the CSF3R clone was depleted, yet the expansion of a NRAS mutated subclone was promoted.

**Conclusion:**

These results demonstrate the usefulness of neutrophil migration analyses to uncover corresponding alterations of neutrophil migration in rare myeloid neoplasms. Furthermore, in addition to monitoring migration the determination of morphological features of live neutrophils might represent a useful tool to monitor the effectiveness of therapeutic approaches.

## Background

Migration assays of neutrophil granulocytes, referred to as neutrophils, bear strong potential as valuable diagnostic and surveillance tools. As neutrophils are among the first cells to counter infections [1], altered neutrophil migration patterns were observed during acute and chronic conditions, such as sepsis [2, 3], asthma [4], chronic inflammatory bowel disease [5] or multiple sclerosis [6]. When recruited into inflamed tissue, neutrophils do rely on their ability to autonomously migrate towards the area of infection. Chemotactic stimuli attracting neutrophils are e.g. secreted pathogenic peptides containing formyl-methionine (fM) or CXCL cytokines, released by patrolling immune cells or endothelial cells in contact with the pathogens [7, 8]. Therefore, neutrophil migration is rapidly altered in response to ongoing inflammation. Additionally, neutrophil migration is altered in cancer. Reports showed altered neutrophil migration in human head and neck cancer as well as small lung cell carcinoma [9–11]. These findings were corroborated in a number of murine cancer models revealing increased neutrophil motility [12]. However, despite the large body of evidence for the importance of motility for neutrophil function and its modification in disease states, there have been limited attempts to exploit this knowledge for the diagnosis of human diseases.

In this regard, we recently demonstrated that neutrophil migration strongly correlates with the revised international prognosis scoring system (IPSS-R) in myelodysplastic syndrome [13], representing a neoplasia affecting neutrophil functions such as degranulation and phagocytosis [14]. Here, neutrophils from severe MDS cases, with a high risk of blast transformation, displayed significantly lower migration speed than lower-risk MDS cases or neutrophils from healthy donors. Furthermore, the recovery of normal migration patterns during therapy correlated with a successful response to the treatment, pointing at the analysis of migration as a potential monitoring tool for therapy [13].

Atypical chronic myeloid leukemia (aCML) is a rare subtype of myelodysplastic / myeloproliferative neoplasms (MDS/MPN) characterized by poor prognosis and lack of standardized treatment algorithms [15, 16]. aCML mostly manifests in elderly patients with male predominance [17]. It is characterized by elevated white blood cell (WBC) counts, mainly due to increased granulocyte numbers, splenomegaly and severe dysgranulopoiesis with abnormal chromatin clumping [17, 18]. As all MDS/MPN subtypes, aCML lacks distinct genetic alterations facilitating the diagnosis [17]. However, aCML cases have been reported to be associated with mutations of spliceosome proteins, e.g. in U2AF1, of epigenetic modifiers, e.g. in ASXL1, TET2, EZH2, and of signaling molecules, e.g. in NRAS, KRAS, JAK2, CSF3R [19]. Especially the role of mutations in the CSF3R gene, coding for the G-CSF receptor, in aCML were controversially discussed as CSF3R mutations are defining mutations in the diagnosis of chronic neutrophilic leukemia (CNL) [20, 21]. However, studies also reported CSF3R mutations in aCML [22]. Hence, CSF3R mutations represent one of the overlapping features between CNL and aCML [19, 23]. Most CSF3R mutations found in human leukemias involve either truncations or membrane proximal mutations [22]. The CSF3R p.T618I mutation diagnosed in the case of the present study is a membrane proximal mutation causing ligand-independent activation of the down-stream signaling JAK/STAT pathway involving predominantly JAK1/2 in turn leading to unchecked neutrophil proliferation [23]. In this circumstance, the JAK1/2 inhibitor ruxolitinib has been reported as a potential effective CNL therapeutic option [24, 25]. In light of these promising reports, ruxolitinib treatment of CNL and aCML patients with CSF3R mutations is currently investigated within clinical trials (NCT02092324).

As the diagnosis of aCML is a complex endeavor and criteria for the monitoring of aCML therapy have not been established, we investigated the applicability of our novel migration assay in this disease setting.

Here, we analyzed neutrophil migration of an aCML patient in a longitudinal manner. Neutrophils of the present aCML case displayed severely reduced migration compared to healthy controls. Upon treatment with ruxolitinib, neutrophil migration remained at a low level, even though blood parameters and clinical presentation of the patient improved. Interestingly, before initiating ruxolitinib treatment, aCML neutrophils had a flattened morphology steadily normalizing upon treatment with ruxolitinib.

Our findings suggest that analyses of neutrophil migration and morphology might be a valuable diagnostic / monitoring tool for myeloid neoplasms in general. Hence, we conclude that neutrophil migration analyses may be suitable to monitor a spectrum of hematological diseases and should possibly be part of future diagnostic workup strategies and therapy monitoring.

## Methods

### Blood samples

Healthy controls were provided by the Institute for Medical Informatics, Biometry and Epidemiology (IMIBE), University Hospital Essen, Essen, Germany, as part of the Heinz-Nixdorf Recall MultiGeneration (HNRM) study. This study served to extend the Heinz-Nixdorf Recall Study (HNRS), whose objectives and study design were published previously [26]. Both studies were approved by the responsible institutional ethics committees and followed strict internal and external quality assurance protocols. Written informed consent was obtained from all participants. In this manuscript, *n* = 11 of the overall analyzed *n* = 111 blood samples were included to match the presented aCML case in age and gender (*n* = 6) or age only (*n* = 5) (Table 1). Blood of the indicated patient suffering from atypical chronic myeloid leukemia (aCML) was drawn within the out- and in-patients units of the Department of Hematology (University Hospital, Essen, Germany) after written informed consent was obtained. All blood samples were obtained in EDTA-supplemented tubes and transported for 30 min up to 1 h in a VACUETTE® transport container (VTC) (Greiner Bio-One, Kremsmünster, Austria) according to the UN 3373 regulation.

**Table 1:** Basic information on aCML patient and the age- and gender or age-matched controls analyzed in this study

	Controls (migration)	Controls (flow cytometry)	aCML
Individuals	6	5	1
Age [y] (median, range)	69 (66–74)	72 (68–74)	69
Sex (m:f)	6:0	2:3	male

### Next-generation sequencing

To detect somatic, mutational events, a molecular screen was set up analyzing 65 candidate genes in unseparated patient leukocytes derived from peripheral blood samples taken soon after 1st diagnosis, at follow-up-1 after ruxolitinib (sample taken 7 months after start of treatment) and follow-up-2 (sample taken 12 months after start of treatment). DNA of unenriched leukocytes from these consecutive samples was analyzed by next generation gene capture based deep sequencing (NGS) using a custom myeloid gene panel (Agilent SureSelect QXT, target enrichment protocol for loci ABL1 (E4–11), ARID1A, ASXL1 (E12), ATRX (E8_10, 17_35), BCOR, BCORL1, BRAF (E15), CALR (E9), CBL (E8,9), CLBB (E9,10), CBLC (E7), CEBPA, CSF3R (E13–17), CSMD1, CSNK1A1 (E3,4), CUX1, DNMT3A, EED, ETNK1, ETV6, EZH2, FLT3 (E14–15,20), GATA1, GATA2, GNAS (E7–9), HRAS, IDH1 (E4), IDH2 (E4), IKZF1, JAK2 (E12–16), JAK3, KIT (E2,8–17), KDM6A (syn. UTX), KMT2A (syn. MLL), KRAS, MPL (E4–12), NPM1 (E12), NRAS, PDGFRA (E12,14,18), PHF6, PIGA, PRPF40B, PTEN (E5,7), PTPN11 (E3,13), RAD21, RUNX1, SETBP1 (E4), SF1, SF3A1, SF3B1 (E13–16), SH2B3 (E2), SMC1A (E2,3,10-12,16–18), SMC3, SRSF2 (E1), STAG1, STAG2, STAT3 (E3,21), SUZ12 (E10–16), TET2, THPO, TP53, U2AF1 (E2,6), U2AF2, WT1 (E7,9), ZRSR2, coding exons +/− 20 bp, „E “denotes exon) on an Illumina MiSeq platform. The sequencing runs yielded 2.4 to 3.5 million reads for the samples with totals of 4.7 to 10.6 gigabases in the untrimmed raw data of the sequencing runs, whereof 91.9, 95.6, and 94.7% had quality scores exceeding Q30, resulting in average coverages of 857, 823, and 1042 reads per base, respectively. The LOD for somatic mutations varies depending on mutation type, percentage of neoplastic cells in the sample and copy number of individual loci. Mostly, mutations with VAF > 4% can be detected with our bioinformatics pipeline: Bioinformatics and evaluation of sequence data after cutadapt Version: 1.9.1, bwa Version: 0.7.5a-r405, SAMtools Version: 1.2 (using htslib 1.2.1). Software: Seqnext (JSI) Version 4.3.1; if required for confirmative Sanger: Seqpilot (JSI) Version 4.4.0 Analyzed NGS data after trimming were 100% above QS-cutoff > 30 (mostly ≥38). The ROI were 100% over minimal sequencing deepness of 100. Mutation nomenclature according to HGVS. Reference sequences of genes in which mutations were detected are given in italics: ASXL1_NM_015338_c.1934dup, p.Gly646Trpfs*12; CSF3R_NM_000760_c.1853C > T p.Thr618Ile plus presumably germline variant CSF3R_ NM_000760_c.1795C > A, p.His599Asn; TET2_NM_001127208_c.3320C > G p.Ser1107Ter; TET2_ NM_001127208_c.4222G > T p.Gly1408Ter; CEBPA_NM_04364_c.1004 T > A p.Leu335Gln; EZH2_NM_004456_c.2069G > A p.Arg690His; NRAS_NM_002524_c.35G > A p.Gly12Asp; STAG2_NM_001042749_c.1178 T > A p.Leu393Ter; U2AF1_NM_006758_c.460 T > A p.Cys154Ser.

### Neutrophil isolation and migration assay conditions

For all healthy controls, neutrophils were isolated from 3 ml EDTA-supplemented blood via density centrifugation using Polymorphprep™ (Cat. No.: 1114683, AXIS-SHIELD, Oslo, Norway) as previously described [13]. In short, Polymorphprep™ was overlaid with blood at a 1:1 ratio and centrifuged at 450 rcf for 30 min without brake. Polymorphonuclear cells (PMN) were collected and washed with sterile PBS (Cat. No.: P04–36500, PAN-Biotech, Aidenbach, Germany). Erythrocytes were lysed for 10 min at room temperature (RT) in lysis buffer, containing 155 mM NH_4_Cl, 10 mM KHCO_3_, 0.1 mM EDTA in distilled H_2_O. After another washing step in sterile PBS, cells were resuspended in sterile hematopoietic progenitor growth medium (HPGM, Cat. No.: PT-3926, Lonza, Basel, Switzerland) and automatically counted using a Cellometer Auto T4 (Nexcelom Bioscience, Lawrence, MA, USA). Since Polymorphprep™ isolation did not reliably separate neutrophils from the aCML patient, isolations of aCML neutrophils after day 14 were carried out using magnetic negative isolation with the MACSxpress® Neutrophil Isolation Kit (Cat. No.: 130–104-434, Miltenyi Biotec, Bergisch Gladbach, Germany) according to manufacturer’s instructions. Residual erythrocytes were also magnetically depleted using MACSxpress® Erythrocyte Depletion Kit (Cat. No.: 130–098-196, Miltenyi Biotec) according to manufacturer’s instructions. Afterwards, purified neutrophils were washed in sterile PBS, resuspended in sterile HPGM and automatically counted. Comparability of the procedures was ensured by side-by-side measurements of the same sample on day 14 (Supplemental Figure 2A) and as previously detailed [13]. The neutrophil migration assay was performed as previously described [13]. Briefly, purified neutrophils were seeded in a 96 Well μ-Plate (Cat. No.: 89621, ibidi, Martinsried, Germany) at a density of 8250 cells per well (growth area: 0.56 cm^2^) in 198 μl HPGM supplemented with serum replacement 3 (SR3, final concentration: 0.3x, Cat. No.: S2640, Sigma-Aldrich, Munich, Germany). Neutrophils were stimulated with 2 μl fMLP (final concentration: 10 nM; Cat. No.: F3506, Sigma-Aldrich, Munich, Germany), 2 μl human recombinant CXCL1 (final concentration: 100 ng/ml; Cat. No.: 275-GR-010/CF, R&D Systems, Minneapolis, MN, USA), or 2 μl human recombinant CXCL8 (final concentration: 100 ng/ml; Cat. No.: 208-IL-010/CF, R&D Systems). As all stimuli were reconstituted in sterile PBS, the addition of 2 μl PBS alone served as a vehicle control. The plates were centrifuged and incubated at 37 °C, 5% CO_2_ for 20 min before microscopy.

### Time-lapse microscopy and auto-tracking

All samples were imaged in a Leica DMI6000 B (Leica Microsystems, Wetzlar, Germany) coupled to a workstation running Leica Application Suite X (LASX, Leica Microsystems) with a motorized stage with a HC PL FLUOTAR L 20x/0.40 DRY objective (Cat. No.: 11506243, Leica Microsystems) at an imaging rate of one frame every 8 s for 1 h at 37 °C, without CO_2_. The generated movies were exported as *.mov files. These files were analyzed with the Automated Cellular Analysis System (ACAS, Metavi-Harmony software, MetaVi Labs, Austin, TX, USA; sales@metavilabs.com). The evaluation interval was set to 30 s, the minimum track duration to 60 s, the movement threshold to 8 μm and the microscopy resolution to 0.458716 pixel/μm.

### Flow cytometry

One hundred thousand purified neutrophils were stained with the following antibodies: CD15 VioBlue (dilution: 1:100, clone: VIMC6, Cat. No.: 130–113-488, Miltenyi Biotec), CD16 FITC (dilution: 1:100, clone: REA423, Cat. No.: 130–113-392, Miltenyi Biotec), fMLP receptor Alexa Fluor 647 (final dilution: 1:100, clone: 5F1, Cat. No.: 565623, BD Biosciences, San Jose, CA), CXCR1 PE (dilution: 1:100, clone: 8F1, Cat. No.: 130–105-352, Miltenyi Biotec), and CXCR2 PE-Vio770 (dilution: 1:20, clone: REA208, Cat. No.: 130–100-930, Miltenyi Biotec). After an incubation step of 15 min in the dark at 4 °C, the suspensions were diluted 1:1 with PBS and analyzed on a MACSQuant VYB (Miltenyi Biotec).

### Manual cell size analysis

To quantify the changes in cellular morphology of aCML neutrophils and neutrophils from healthy donors, the size of cells in the respective video were manually analyzed using ImageJ (Rasband, W.S., ImageJ, U. S. National Institutes of Health, Bethesda, Maryland, USA, https://imagej.nih.gov/ij/, 1997–2017.). For that, the first image of every video was exported as *.tif from the LASX software and imported to ImageJ. Subsequently, the outer cell margins were manually marked as regions of interest (ROI) and the occupied area was computed by ImageJ’s ROI manager. Results were given in μm^2^. Additionally, five age- and gender-matched probands were quantified as controls.

### Statistical analysis

All statistical analyses were performed using GraphPad Prism™ (Version 6.07, GraphPad Software, San Diego, CA, USA). Experimental data were plotted as bar graphs or scatter dot plots. Statistical computation, such as computation of *p*-values and others, was performed as described in the respective figure legends.

## Results

### Clinical presentation of the aCML case

**Fig. 1:**
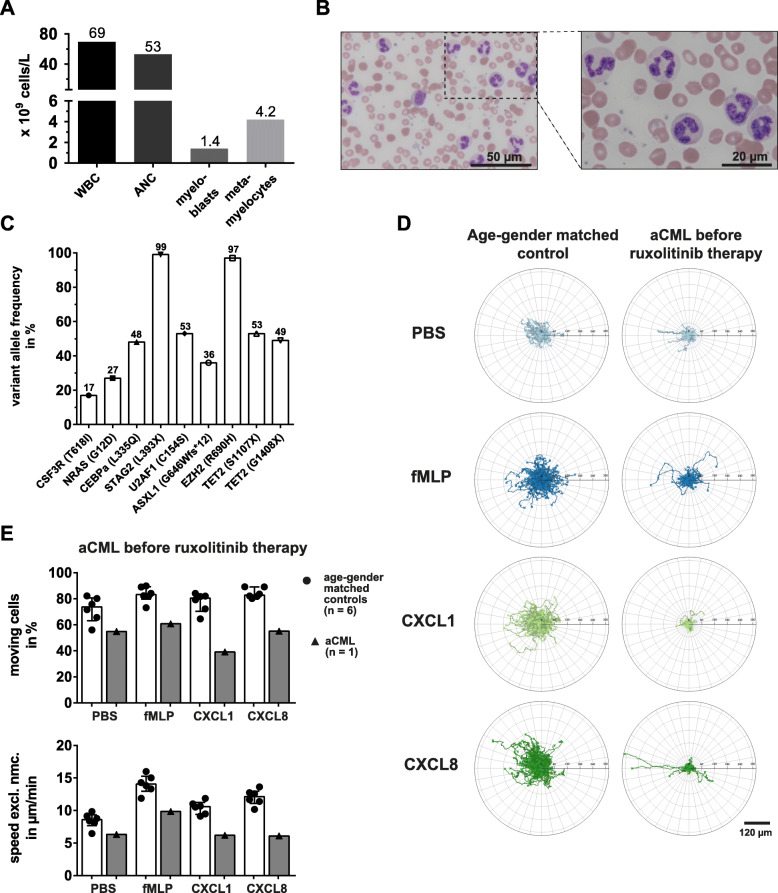
Migration of aCML neutrophils is severely impaired. **a** Peripheral blood parameters of the untreated aCML patient. **b** Microscopic images of peripheral blood smear from a 69-year old, untreated aCML patient. 50x magnification (top) and 100x magnification (bottom) are shown. **c** Variant allele frequency (VAF) of mutated candidate genes in unseparated patient peripheral blood leukocytes. **d** Representative trajectory plots of migrating neutrophils from an age- and gender-matched control (left) and the aCML patient before therapy (right). From top to bottom, the cells were treated with PBS as a vehicle control, fMLP [10 nM], CXCL1 [100 ng/ml], and CXCL8 [100 ng/ml] in vitro, continuously imaged for 1 h under a widefield microscope and single cells were automatically tracked. **e** Statistical summary of percentage of moving cells (top) and speed excluding non-moving cells (speed excl. Nmc., bottom) of the untreated aCML patient (black triangles, grey bars) and the age- and gender-matched controls (black dots, white bars; *n* = 6). Each symbol represents a single individual. Bars are given as median ± interquartile range

A 69-year-old male with a four-month history of the suspected diagnosis of a myelodysplastic/myeloproliferative neoplasm was referred to our department. Despite cytoreductive therapy with hydroxyurea, the patient presented with a white blood cell (WBC) count of 69 × 10^9^/L and an absolute neutrophil count (ANC) of 53 × 10^9^/L (Fig. 1a). The hemoglobin and platelet counts were 10.2 g/dl and 367 × 10^9^/L, respectively. The manual differential revealed 76% neutrophils, 2% band forms, 6% metamyelocytes, 3% myelocytes and 2% myeloblasts. Dysgranulopoiesis with hypogranularity, abnormal chromatin clumping, Pelger-Huët anomaly and multiple nuclear projections was observed (Fig. 1b). A bone marrow biopsy and aspirate revealed myeloid hyperplasia without increased blasts and without reticulin fibrosis. Cytogenetics did not show abnormalities and the BCR-ABL1 PCR and FISH diagnostics for PDGFRA, PDGFRB and FGFR1 rearrangements were negative. The next generation sequencing analysis revealed the presence of mutations in eight genes including the CSF3R p.T618I mutation (Fig. 1c). Within the exons of the other genes included in the panel no mutations were identified. According to the revision of the World Health Organization classification of myeloid neoplasms 2016 [16], CSF3R mutations are strongly associated with chronic neutrophilic leukemia (CNL), however do also appear in atypical chronic myeloid leukemia (aCML). The NRAS as well as the TET2, ASXL1 and EZH2 mutations were frequently observed in aCML [27]. Given the presence of dysgranulopoiesis in combination with neutrophil precursors > 10%, the patient was diagnosed with aCML. Hematopoietic stem cell transplantation as a treatment option was deferred due to advanced age and chronic kidney disease. Even though the patient was on hydroxyurea, the WBC count and constitutional symptoms were poorly controlled. Because of the potential benefit of ruxolitinib in CSF3R T618I mutated myeloid neoplasms [25], the patient was commenced with an off-label prescription of ruxolitinib with a dose of 10 mg twice daily (day 0).

### aCML neutrophil migratory impairment

The migration of neutrophil in response to fMLP is highly reduced in individuals suffering from severe MDS [13]. To test, whether this functional impairment is also present in other myeloid neoplasms, we assessed the migratory capacity of neutrophils in the described case of aCML. Interestingly, before therapy and in contrast to cells from age-matched healthy controls, aCML neutrophils were almost completely unresponsive to fMLP, CXCL1 and CXCL8 (Fig. 1d). In fact, automated tracking analysis revealed that already the baseline percentage of moving cells and their speed were reduced in aCML neutrophils (54.9%, 6.33 μm/min) compared to control values (71.90 ± 4.01%, 8.457 ± 0.47 μm/min) (Fig. 1e). Moving cells and speed upon fMLP treatment only reached values of 60.8% and 9.85 μm/min, as opposed to 83.38 ± 2.47% and 14.06 ± 0.58 μm/min for healthy neutrophils. Moving cells were most severely reduced under CXCL1 treated conditions, with 39.1% versus 77.28 ± 3.06% in healthy blood donors. CXCL1 treated aCML neutrophils only reached a mean speed of 6.19 μm/min, which was lower than the speed reached by CXCL1-triggered control neutrophils (10.45 ± 0.41 μm/min) or even aCML neutrophils speed under non-stimulated (PBS) conditions. Likewise, migrating cells and speed were diminished upon CXCL8 treatment (55.1%, 6.09 μm/min) compared to healthy neutrophils (84.33 ± 1.59%, 12.03 ± 0.49 μm/min, Fig. 1e). Taken together, neutrophils from this aCML case displayed impaired migration comparable to the previously published finding of neutrophils from MDS patients [13].

### Increment of aCML neutrophil size

**Fig. 2:**
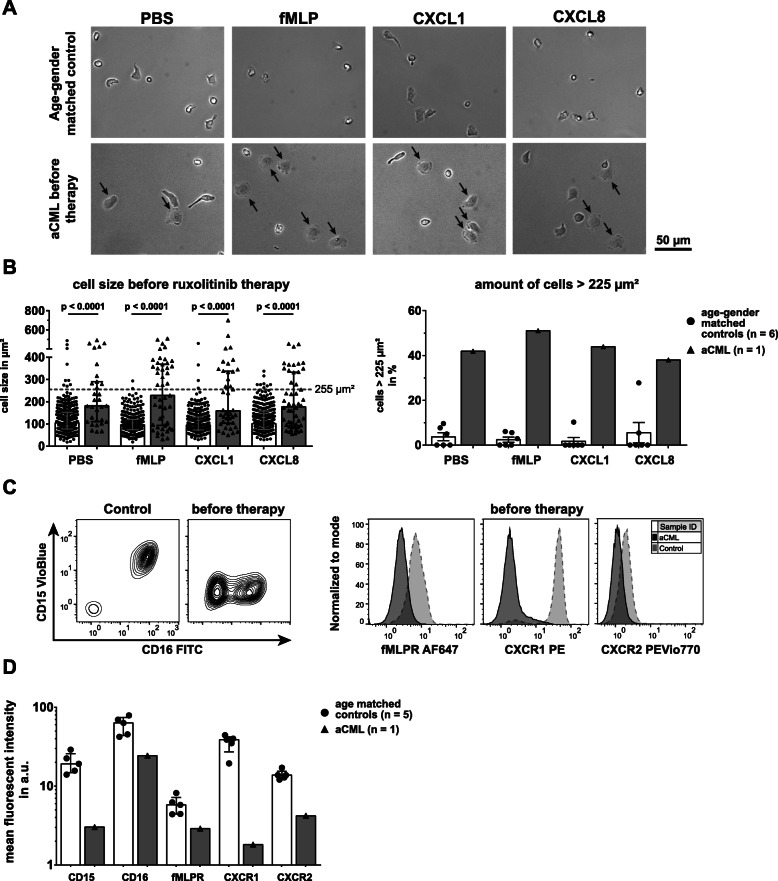
aCML neutrophils show distinct morphologic changes and reduced expression of surface CD16, CD15, fMLPR, CXCR1 and CXCR2. **a** The first frame of image sequences acquired during video microscopy of neutrophils from an age- and gender-matched control (top) and the aCML patient before therapy (bottom). From left to right, the cells were treated with PBS as a control, fMLP [10 nM], CXCL1 [100 ng/ml] and CXCL8 [100 ng/ml]. Black arrows in the lower panel indicate prominently enlarged cell bodies. Magnification: 20x. **b** Statistical summary of the cell size in μm^2^ of aCML neutrophils before therapy (left) and the relative number of neutrophils with a cell size of > 225 μm^2^ (right). Both parameters were compared to age- and gender-matched controls (n = 6). On average, 41 and 56 cells per condition were analyzed in age- and gender-matched controls and the aCML patient, respectively. Bars are given as median ± interquartile range and the given *p*-values were calculated using Mann-Whitney *U* test. The cutoff of 225 μm^2^ (grey dashed line, left) was chosen as assuming a perfect circle equals a diameter of 16 μm and is thus close to a neutrophil’s normal diameter in cell culture [29]. **c** Representative contour plots and histograms of purified neutrophils. Analyses of CD16 (FITC) and CD15 (VioBlue) (left) and fMLPR, CXCR1 and CXCR2 (right) expressions are shown. An age- and gender-matched control (control, left of left panel; dotted light grey line of right panel) and aCML neutrophils before ruxolitinib therapy (before therapy, right of left panel; solid dark grey line of right panel) are depicted. **d** Statistical summary of expression levels for CD16, CD15, fMLPR, CXCR1 and CXCR2 on purified neutrophils from age- and gender-matched controls (controls; black dots, white bars; *n* = 5) and aCML neutrophils before treatment (before therapy; black triangles, grey bars; *n* = 1). Expression levels are given as the mean fluorescent intensity (mfi) and bars are given as median ± interquartile range

The shape and level of adherence of a cell crucially influences its migration [28]. In our migration assay, healthy neutrophils were characterized by low adhesion and hence a compact migratory shape [13] (Fig. 2a). In contrast, a large portion of aCML neutrophils were abnormally shaped and more flattened (Fig. 2a, black arrows). The mean cell area of aCML neutrophils was significantly higher compared to control neutrophils (Fig. 2b**,** left panel). To quantify the number of enlarged cells, we plotted the distribution of the cell size of healthy and aCML neutrophils for all conditions (Supplemental Figure 1A, representative distribution, here CXCL1 treatment). In healthy controls, we found a dramatically lower proportion of larger, flattened out cells compared to the aCML patient. A greater number of aCML neutrophils had a cell size of over 225 μm^2^ (Fig. 2b**,** right panel).

### Reduced expression of lineage markers and stimuli receptors by aCML neutrophils before ruxolitinib therapy

To elucidate whether defective neutrophil migration might originate from changes in signaling receptor expression, we performed flow cytometry analyses of two neutrophil lineage markers, CD15 and CD16 [30], and the signaling receptors fMLP receptor (fMLPR), CXCR1 and CXCR2. The low affinity IgG receptor FcγRIII (CD16) in its glycosylphosphatidylinositol (GPI)-linked form is expressed on mature neutrophils [31] and important during the secretion of reactive oxidants [32]. CD15 is a carbohydrate antigen present on mature myeloid cells that may be involved in cell-cell contact [33] and adherence [34]. Interestingly, CD15 was absent on aCML neutrophils and the assessment of CD16 revealed an overall reduced expression in comparison to healthy control neutrophils (Fig. 2c, left panel). Additionally, the expression of all signaling receptors was diminished, most prominently for CXCR1, the receptor for CXCL8 (Fig. 2c, right panel). Quantification of mean fluorescent intensity (mfi) of control and aCML neutrophils revealed severely reduced expression of both CD16 and CD15, as well as fMLPR, CXCR1 and CXCR2 (Fig. 2d), suggesting not only alterations in maturation and differentiation of the neutrophils, but abolished expression of key signaling receptors for chemotactic stimuli.

### Effect of ruxolitinib on neutrophil morphology, migration and receptor expression

**Fig. 3:**
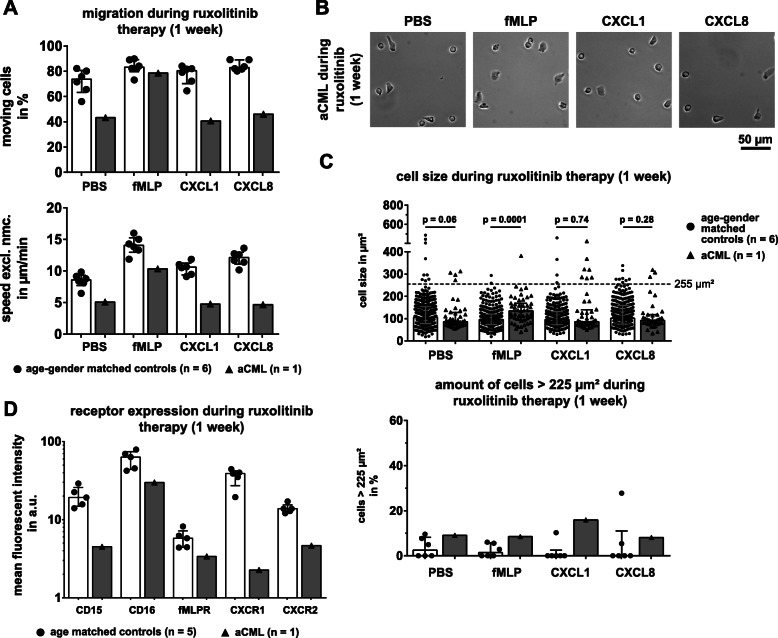
Cell size normalizes already after 1 week of ruxolitinib treatment, but migration and receptor expressions remain unaffected. **a** Statistical summary of percentage of moving cells (top) and speed excluding non-moving cells (speed excl. Nmc., bottom), of purified aCML neutrophils after 1 week of treatment with ruxolitinib (black triangles, grey bars; *n* = 1), compared to the healthy age- and gender-matched controls (black dots, white bars; *n* = 6). Each symbol represents a single individual. Bars are given as median ± interquartile range. **(B)** The first frame of image sequences acquired during video microscopy of neutrophils of the aCML patient after 1 week of ruxolitinib therapy. From left to right, the cells were treated with PBS as a control, fMLP [10 nM], CXCL1 [100 ng/ml] and CXCL8 [100 ng/ml]. Magnification: 20x. **c** Statistical summary of the cell size in μm^2^ of aCML neutrophils (black triangles, grey bars) after 1 week of ruxolitinib treatment (top) and the relative number of neutrophils with a cell size of > 225 μm^2^ (bottom). Both parameters were compared to age- and gender-matched controls (black dots, white bars; *n* = 6). On average, 41 and 56 cells per condition were analyzed in age- and gender-matched controls and the aCML patient, respectively. Bars are given as median ± interquartile range and the given p-values were calculated using Mann-Whitney *U* test. The cutoff of 225 μm^2^ (top) is displayed as a dashed grey line. **d** Statistical summary of expression levels for CD16, CD15, fMLPR, CXCR1 and CXCR2 on purified neutrophils from age-matched controls (controls; black dots, white bars; *n* = 5) and aCML neutrophils after 1 week of ruxolitinib treatment (1 week; black triangles, grey bars; *n* = 1). Expression levels are given as the mean fluorescent intensity (mfi). Bars are given as median ± interquartile range

Next, we analyzed aCML neutrophils 6 days after the onset of ruxolitinib therapy. Relative PBS and CXCL8 stimulated neutrophil migration dropped, but rose for fMLP and CXCL1, as compared to migration before therapy (Fig. 3a, upper panel). aCML neutrophil baseline (PBS) speed as well as speed under CXCL1 and CXCL8 decreased compared to the speed before initiating ruxolitinib treatment (Fig. 3a, lower panel). aCML migration speed upon fMLP treatment was unaltered after 1 week of ruxolitinib (9.9 μm/min vs. 10.3 μm/min). In contrast, aCML neutrophils changed after ruxolitinib treatment. The cells appeared rounder and smaller compared to their morphology before therapy (Fig. 3b). The cell size of neutrophils decreased compared to before therapy and was indistinguishable from healthy neutrophils under CXCL1 and CXCL8 stimulated conditions (Fig. 3c**,** upper panel). Cell size of aCML neutrophils upon fMLP treatment was still significantly higher (*p* = 0.0001). The number of neutrophils with a cell size > 225 μm^2^ dropped for all stimulation conditions (Fig. 3c, lower panel) compared to the size before treatment. Only 9.1% of all cells were still enlarged when incubated with PBS after 1 week of ruxolitinib treatment, as opposed to 41.9% before therapy. The percentage of flattened cells upon fMLP, CXCL1 and CXCL8 treatment decreased as well.

The reduced expression of CD16, CD15, fMLPR, CXCR1 and CXCR2 increased slightly after 1 week of treatment (Fig. 3d). The mfi of the investigated receptors rose to 4.5 (CD15), 29.9 (CD16), 3.38 (fMLPR), 2.27 (CXCR1) and 4.65 (CXCR2), compared to before onset of the ruxolitinib therapy. However, the expression levels still remained far below average levels of healthy, age-matched controls.

### Impact of long-term ruxolitinib treatment on clinical presentation, CSF3R p.T618I mutational burden and neutrophil migratory parameters

**Fig. 4:**
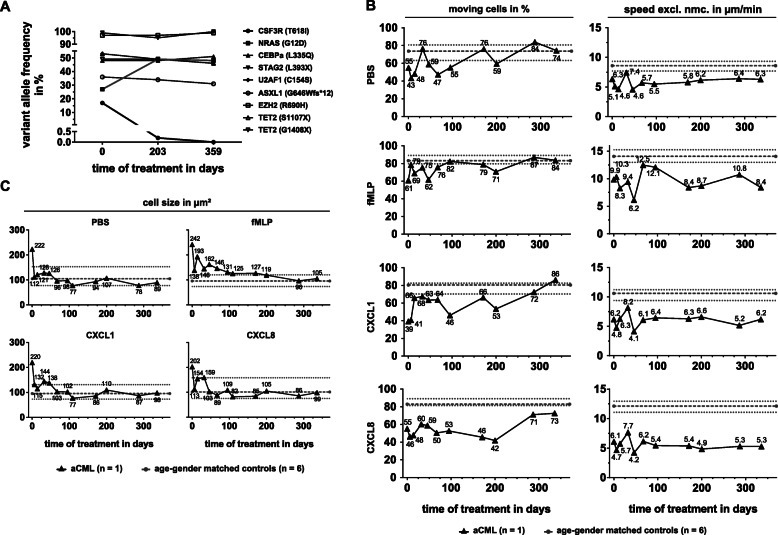
Ruxolitinib therapy causes loss of CSF3R mutated clone, cell size normalization, but only marginally compensates the migration defect of aCML neutrophils. **a** Variant allele frequency (VAF) of candidate genes of the aCML patient over the course of ruxolitinib therapy. **b** Changes in the migratory patterns, percentage of moving cells (left) and speed excluding non-moving cells (speed excl. Nmc., right), of the aCML neutrophils over the course of treatment. From top to bottom, cells were stimulated with PBS, fMLP [10 nM], CXCL1 [100 ng/ml] and CXCL8 [100 ng/ml]. Black triangles and black solid lines indicate aCML neutrophils (every timepoint *n* = 1), while grey dots and grey dashed lines indicate the median and the grey dotted lines indicate the interquartile range of the age- and gender-matched controls (*n* = 6). Numbers label the specific values of aCML neutrophils reached for percentage of moving cells (left) and speed excluding non-moving cells (right) respectively. **c** Changes in cell size of aCML neutrophils under the four different stimulation conditions over the course of therapy. Black triangles and black solid lines indicate aCML neutrophils (every timepoint *n* = 1), while grey dots and grey dashed lines indicate the median and the grey dotted lines indicate the interquartile range of the age- and gender-matched controls (*n* = 6). On average, 41 and 56 cells per condition were analyzed in age- and gender-matched controls and the aCML patient, respectively. Numbers label the specific cell sizes as the mean cell size of all cells

Treatment with ruxolitinib and hydroxyurea caused the WBC to drop from 69 to 16 × 10^9^ cells/L, but when hydroxyurea was discontinued, the WBC count rapidly rose to 116 × 10^9^/L (day 45) (Supplemental Figure 3A). Ruxolitinib was increased (20 mg, twice daily) and hydroxyurea restarted, causing the WBC to drop again (day 73). However, WBC rose again when the patient was admitted to hospital because of sinusitis with fever and bleeding of a gastric ulcer. Two months later, the WBC was rising again with an increasing percentage of myeloblasts (22%, day 193). Strikingly, the next generation sequencing revealed that, at day 203, the size of the CSF3R T618I mutated clone was barely detectable while the NRAS mutated clone rose from 27 to 49% VAF (Fig. 4a). This remained stable up to day 359. Cytoreductive treatment with hydroxyurea was complemented by mercaptopurin and the WBC count dropped below 10 × 10^9^ cells/L with a blast percentage less than 5%. The patient was again admitted to hospital with pneumonia and hemoptysis (day 256). In the subsequent weeks, the diagnosis of pulmonary mucormycosis was made. The patient died despite treatment with voriconazole, liposomal amphotericin B and isavuconazole (day 370).

Shortly after onset of ruxolitinib therapy, the percentage of moving cells almost reached normal levels for all migration stimuli except for CXCL8 (Fig. 4b, left panel). Interestingly, the proportion of moving cells differed between the timepoints early in therapy. Migration speed never fully recovered to normal levels for any of the stimuli, but especially migration after CXCL8 triggering remained severely impaired over the course of disease (Fig. 4b, right panel). On the other hand, the cell size normalized rapidly after onset of ruxolitinib therapy and remained mostly stable during the observed period (Fig. 3c). Only during fMLP stimulation, the cell size remained elevated for a prolonged time, before returning to normal levels after day 200. For PBS, CXCL1 and CXCL8 treatment, the number of neutrophils with a cell size of > 225 μm^2^ reached normal levels after day 46–67 but remained increased for fMLP treatment during the whole observation period (Supplemental Figure 1B).

The expression levels of CD15 and CD16 increased until day 109, after which the expression levels decreased sharply (Supplemental Figure 2B). At the end of our observations, CD16 expression had returned to normal levels of the age-matched controls, while CD15 expression remained reduced. However, fMLPR, CXCR1 and CXCR2 expression remained severely impaired, especially for CXCR1, which was all, but absent from the cells over the whole observation period. As published before, the expression of the signaling receptors does not correlate with migration upon stimuli treatment [13]. We therefore correlated the expression of fMLPR, CXCR1 and CXCR2 on aCML and control neutrophils with the migration upon fMLP, CXCL1 and CXCL8 treatment (Supplemental Figure 2C – F). Except for fMLPR, none of the receptor levels on neutrophils from the aCML patient correlated with the migration behavior of the cells in vitro (Supplemental Figure 2C).

As the presence of immature neutrophils can severely impact the results gained from cell migration assays, we correlated the peripheral blood counts against the migration patterns upon stimuli treatment (Supplemental Figure 3F – H). Interestingly, the percentage of moving cells (Supplemental Figure 3F, first panel) and speed excluding non-moving cells (second panel) upon PBS treatment correlated negatively with the number of leukocytes in the blood. Additionally, the amount of metamyelocytes and banded neutrophils also correlated negatively with the speed upon PBS treatment (Supplemental Figure 3F). When the cells were treated with the stimuli, this correlation was lost for all conditions except for the speed upon CXCL1 and CXCL8 treatment and the amount of metamyelocytes (Supplemental Figure 3G + H).

## Discussion

Neutrophil migration is a promising novel functional parameter to identify states of disease in humans. To elucidate the applicability of our neutrophil migration assay [13] and to determine whether additional migration or morphological parameters in neutrophil migration assays are useful, we studied a single patient suffering from the rare neoplasia aCML.

We have shown that migration of aCML neutrophils remained diminished over the whole observation period. Furthermore, the expression of the low affinity IgG receptor FcγRIII, CD16, and carbohydrate antigen, CD15, which are normally strongly expressed by human mature neutrophils, remained below the levels of matched adult controls. CD16 is normally upregulated with increasing neutrophil maturation [35], yet this did coincide with the amount of immature neutrophils in the circulation of the patient. Furthermore, CD15 expression, which is also regulated during neutrophil maturation [36], was largely absent over the complete observation period and did also not correlate with peripheral immature neutrophil levels. Additionally, we observed a severe reduction of the key chemokine receptors fMLPR, CXCR1 and CXCR2 expression levels. This is remarkable as ruxolitinib therapy caused loss of the CSF3R (p.T618I) clone and was successful in reducing both WBC, peripheral neutrophil precursors and other disease manifestations, like night sweats, weight loss and fatigue. In MDS, however, successful treatment was indicated by a normalization of neutrophil migration. Thus, it is conceivable that the migration defect is the result of multiple mutations, not exclusively CSF3R (p.T618I). It is therefore important to consider that the aCML patient presented here harbored a large number of different mutations, which are typically observed in myeloid neoplasms. CCAAT/enhancer binding protein α (CEBPα) is a transcription factor important during granulocyte differentiation. Mutations in this protein generally cause a diminished activity by preventing DNA binding or downstream translation and reduced activity of CEBPα results in differentiation arrest of granulocytes and hyperproliferation of hematopoietic stem cells (HSCs) [37, 38]. The NRAS (G12D) mutation is one candidate that might directly influence neutrophil migration. Ras proteins are proto-oncogenes that are critical for the signal transduction from cell-surface receptors into inner machinery, thereby controlling cell proliferation, differentiation or cell death [39]. NRAS mutations are frequent in human myeloid leukemias and other cancers and the G12D mutation has been described to drive development of chronic MPN in mice [40]. Indeed, oncogenic NRAS is involved in heightened migration in melanoma cell lines and inhibition of NRAS by microRNAs was successful in reducing trans-well migration [41]. There is evidence that a delicate interplay between ERK and p38 MAPK is needed to regulate directed migration [42]. We have recently reported, that over-phosphorylation of the MAPK p38 upon high fMLP stimulation for 1 h correlated with reduced random migration speed in human neutrophils [13]. This might hint at different mechanisms to induce random versus directed migration or variations due to different analysis timepoint. The NRAS (G12D) mutation in human neutrophils might therefore cause defects in random migration by inducing the hyperphosphorylation of downstream targets.

Further studies are needed to determine whether random and directed migration are in fact differentially affected by NRAS (G12D) and whether there are specific mutations that affect neutrophil migration to distinguish MDS or aCML. Additionally, a study recently reported that ruxolitinib itself showed impairing effects on dendritic cell migration by inhibition of Rho-associated coiled-coil kinase (ROCK) [43]. While we cannot rule out that ruxolitinib therapy was partially responsible for the diminished neutrophil migration reported here, we found low migration speed and recruitment before onset of the therapy as well (Fig. 1e), hinting at a cell intrinsic mechanism underlying this phenomenon. Furthermore, especially the relative number of moving neutrophils upon fMLP, CXCL1 and PBS stimulated conditions increased with therapy start. Neutrophil speed however remained heavily impaired.

An interesting difference between the aCML and the MDS samples, was neutrophil morphology. In aCML the altered morphology normalized in concordance with the therapeutic success of ruxolitinib treatment. aCML neutrophils were significantly enlarged compared to healthy controls before therapy but whether this was the result of an enlarged cytoplasm or increased adherence remains unclear. However, neutrophil size did not correlate with the assessed peripheral blood parameters, like myeloblast and metamyelocyte counts (data not shown), ruling out the possibility that neutrophil progenitors in the assay were responsible for these changes. Additionally, neutrophil morphology normalized to healthy levels already 1 week after the initiation of ruxolitinib therapy. In fact, it has been reported that ruxolitinib causes microtubule instability in JAK (V617F) mutant HEL cells by inhibiting JAK2 and STAT3 activity [44] and might thereby change their morphology. Furthermore, it is conceivable that the CSF3R (T518I) mutant was responsible for the abnormal shape or adherence of neutrophil in our assay, which then normalized by clonal depletion as assessed by NGS (Fig. 4a).

Strikingly, we observed severely reduced expression levels of CD15 and CD16, as well as of the corresponding receptors to the chemokines used in this assay, fMLPR, CXCR1 and CXCR2. While we found no direct correlation between the levels of receptor expression and neutrophil migration behavior in healthy donors [13], it is conceivable that especially the severely impaired migration upon CXCL8 treatment might have been caused by the absence of its signaling receptor CXCR1 [45]. However, CXCL8 can also signal via CXCR2 [45], whose expression levels verged on normal over the course of disease but did not impact neutrophil migration when triggered with CXCL8. As CXCL8 is a key molecule required for the recruitment of neutrophils and their successful extravasation from the blood vessel system [46], this disrupted response to CXCL8 might explain, why the patient remained susceptible to bacterial and fungal infections throughout therapy.

## Conclusion

With the case presented here, we provide evidence that the routine assessment of neutrophil migration and receptor expression provides a broader perspective on diseased neutrophils and impact of a treatment on the patient’s cells. We found compelling evidence that cell shape and degree of adherence were changed over the course of ruxolitinib treatment and coincided with the disappearance of specific clones. Furthermore, we noted an increase in neutrophil speed on day 67 to 12.5 μm/min upon fMLP stimulation (Fig. 4b), which corresponded to the bleeding of a gastric ulcer 6 days later, when the patient was admitted to hospital again. We therefore believe that assessment of neutrophil migration might also facilitate the surveillance of patients with a higher risk for infections. Interestingly, our data also demonstrate that aCML neutrophils were indeed still able to overcome their unresponsive state, but seemed to require additional, host-derived activations to migrate in an in vitro assay.

## Supplementary information

**Additional file 1: Figure S1.** Changes in the relative number of neutrophils above cutoff over the course of ruxolitinib therapy. (A) Cell size distribution of neutrophils upon treatment of CXCL1, representative of all stimuli conditions. Neutrophils were grouped according to their measured cell size in μm^2^ and binned from 25 μm^2^ to 725 μm^2^ with a bin width of 50 μm^2^. Relative frequency was computed by dividing the number of cells in a group by the number of cells in the entire image. The dashed grey line indicates the mean of the age- and gender-matched controls (*n* = 6), the blue line indicates the aCML neutrophils and the red dashed line indicates the cutoff of 225 μm^2^. Binning was performed with GraphPad Prism™. (B) Changes in the relative number of neutrophils with a cell size > 225 μm^2^ under the four stimulation conditions over the course of therapy. Black triangles and black solid lines indicate aCML neutrophils (every timepoint *n* = 1), while grey dots and grey dashed lines indicate the median and the grey dotted lines indicate the interquartile range of the age- and gender-matched controls (*n* = 6). On average, 41 and 56 cells per condition were analyzed in age- and gender-matched controls and the aCML patient, respectively. Numbers label the specific percentage of cells > 225 μm^2^ for the indicated timepoint.

**Additional file 2: Figure S2.** Expression of surface markers of aCML neutrophils over the course of ruxolitinib therapy and correlation with migration patterns. (A) Comparison of CD15 and CD16 expression of aCML neutrophils after 2 weeks of ruxolitinib treatment (2 weeks Ruxo) after two different purification methods: density gradient centrifugation (density gradient, left) and negative magnetic isolation via MACSxpress® separation (MACS, right). **(B)** Changes in CD16, CD15 and fMLPR (top) as well as CXCR1 and CXCR2 (bottom) expression given as mean fluorescent intensity (mfi) of aCML neutrophils over the course of therapy. Black triangles and black solid lines indicate the aCML patient (every timepoint *n* = 1), while grey dots and grey dashed lines indicate the median and the grey dotted lines indicate the interquartile range of the age-matched controls (*n* = 5). Numbers label the specific values for receptor expression at the respective timepoint. **(C-F)** Correlation of receptor expression against migration upon corresponding stimulus treatment. Black triangles indicate the aCML patient (*n* = 1) and grey dots indicate age-matched controls (*n* = 5). Correlations were computed using GraphPad Prism™. Spearman *r* and *p*-value for the correlation of aCML samples are given. (C) fMLPR expression correlated against migration upon fMLPR treatment. (D) CXCR1 expression correlated against migration upon CXCL8 treatment. (E) CXCR2 expression correlated against CXCL1 treatment. (F) CXCR2 expression correlated against CXCL8 treatment.

**Additional file 3: Figure S3.** Changes in leukocyte parameters during ruxolitinib therapy and correlation of peripheral leukocyte counts with migration patterns. (A) – (E) Time course of chosen peripheral blood parameters of the aCML patient over the course of his disease and therapy. Displayed are the absolute WBC (A), myeloblast (B) and metamyelocyte (C) counts in cells/nl as well as the banded neutrophil granulocyte (D) and myelocyte (E) counts relative to the overall WBC in %. Red dots indicate timepoints where migration and flow cytometry data were acquired. (F) Correlation of peripheral cell counts with migration patterns upon PBS stimulation. From left to right: leukocyte counts with moving cells (PBS), leukocyte counts with speed excluding non-moving cells (PBS), metamyelocytes with speed excluding non-moving cells (PBS) and banded neutrophils with speed excluding non-moving cells (PBS). (G) Correlation of metamyelocyte counts with speed excluding non-moving cells upon CXCL1 treatment. (H) Correlation of metamyelocyte counts with speed excluding non-moving cells upon CXCL8 treatment. (F) - (H) Correlations were computed using GraphPad Prism™. Spearman *r* and *p*-values are given for each correlation below the graph.

## Data Availability

The datasets used and/or analyzed during the current study are available from the corresponding author on reasonable request.

## Abbreviations

aCML: Atypical chronic myeloid leukemia
ANC: Absolute neutrophil count
CEBPα: CCAAT/enhancer-binding protein alpha
CNL: Chronic neutrophilic leukemia
CSF3R: Colony stimulating factor 3 receptor
CXCL: Chemokine (C-X-C motif) ligand
EDTA: Ethylenediaminetetraacetic acid
fMLP: N-Formylmethionine-leucyl-phenylalanine
fMLPR: fMLP receptor
mfi: mean fluorescent intensity
GPI: Glycosylphosphatidylinositol
HPGM: Hematopoietic growth medium
IPSS-R: Revised international prognosis scoring system
JAK: Janus kinase
MDS: Myelodysplastic syndrome
MDS/MPN: Myelodysplastic/Myeloproliferative neoplasms
NGS: Next-generation sequencing
NRAS: Neuroblastoma RAS viral oncogene homolog
PBS: Phosphate-buffered saline
PMN: Polymorphonuclear cells
ROCK: Rho-associated protein kinase
ROI: Region of interest
RT: Room temperature
STAT: Signal transducers and activators of transcription
VAF: Variant allele frequency
WBC: White blood cell count
