# Supplementary material for: Defective migration and dysmorphology of neutrophil granulocytes in atypical chronic myeloid leukemia treated with ruxolitinib
Source: BMC Cancer. 2020 Jul 13;20:650. doi: 10.1186/s12885-020-07130-7 (PMC7359613; doi:10.1186/s12885-020-07130-7)

Bornemann et al. Supplemental Figure 1

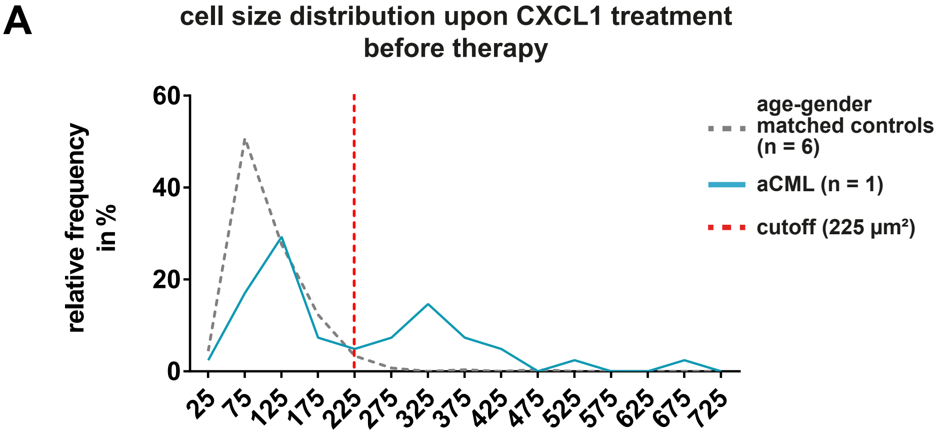

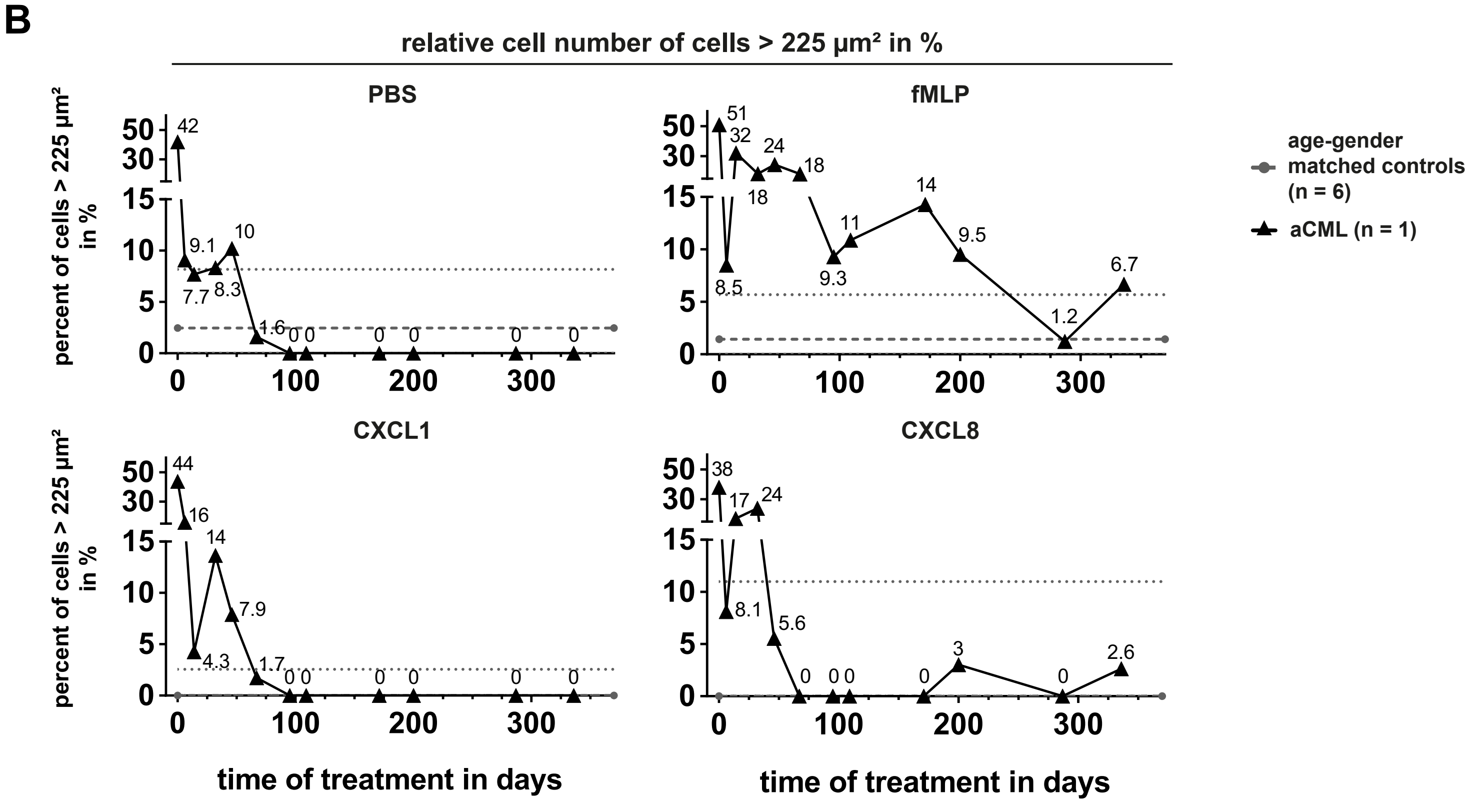

Supplement: Supplementary file 1 — Additional file 1: Figure S1. Changes in the relative number of neutrophils above cutoff over the course of ruxolitinib therapy. (A) Cell size distribution of neutrophils upon treatment of CXCL1, representative of all stimuli conditions. Neutrophils were grouped according to their measured cell size in μm2 and binned from 25 μm2 to 725 μm2 with a bin width of 50 μm2. Relative frequency was computed by dividing the number of cells in a group by the number of cells in the entire image. The dashed grey line indicates the mean of the age- and gender-matched controls (n = 6), the blue line indicates the aCML neutrophils and the red dashed line indicates the cutoff of 225 μm2. Binning was performed with GraphPad Prism™. (B) Changes in the relative number of neutrophils with a cell size > 225 μm2 under the four stimulation conditions over the course of therapy. Black triangles and black solid lines indicate aCML neutrophils (every timepoint n = 1), while grey dots and grey dashed lines indicate the median and the grey dotted lines indicate the interquartile range of the age- and gender-matched controls (n = 6). On average, 41 and 56 cells per condition were analyzed in age- and gender-matched controls and the aCML patient, respectively. Numbers label the specific percentage of cells > 225 μm2 for the indicated timepoint. [file 12885_2020_7130_MOESM1_ESM.pdf]
