# Supplementary figures and images for: Defective migration and dysmorphology of neutrophil granulocytes in atypical chronic myeloid leukemia treated with ruxolitinib
Source: BMC Cancer. 2020 Jul 13;20:650. doi: 10.1186/s12885-020-07130-7 (PMC7359613; doi:10.1186/s12885-020-07130-7)

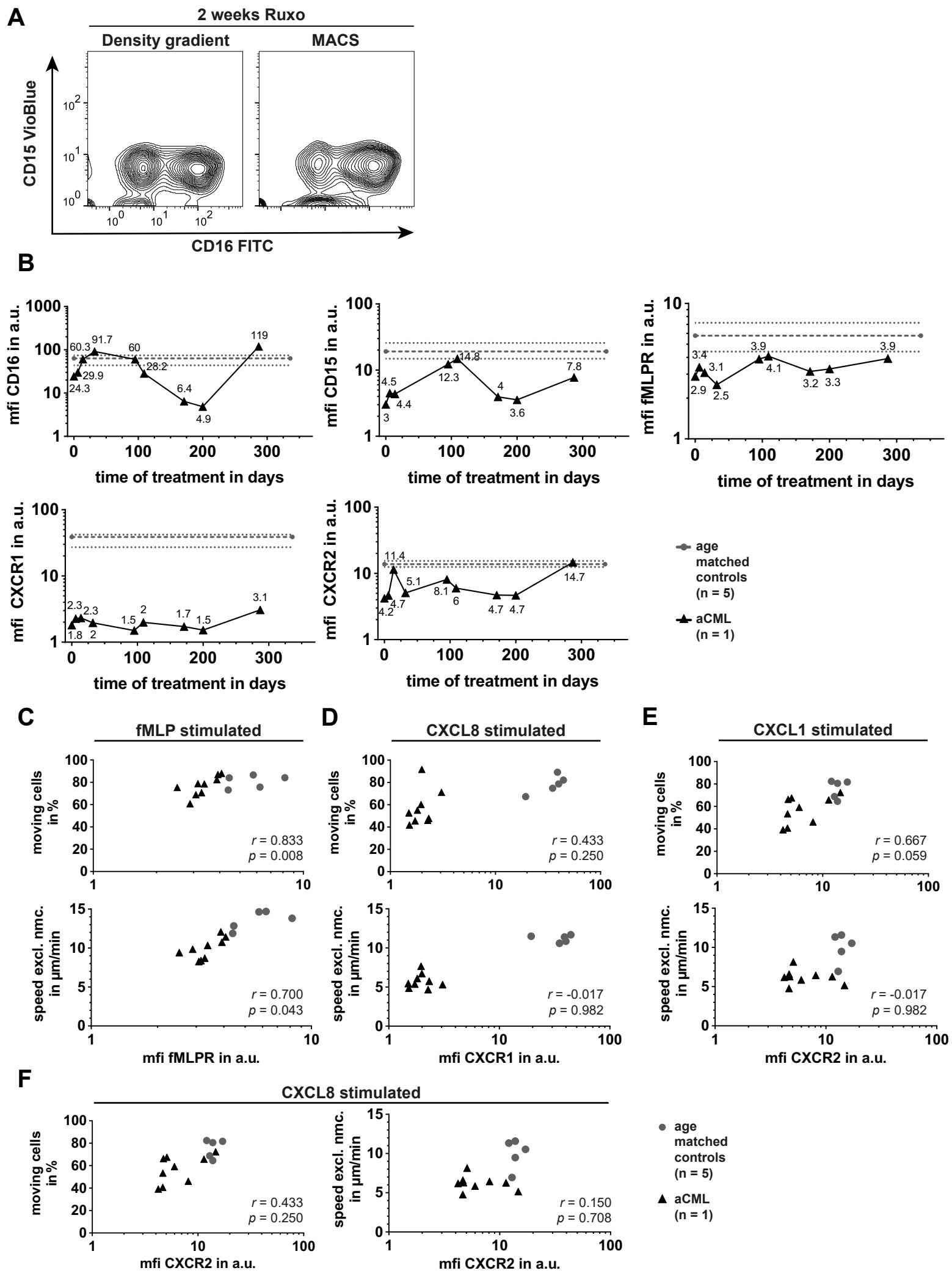

Supplement: Supplementary file 2 — Additional file 2: Figure S2. Expression of surface markers of aCML neutrophils over the course of ruxolitinib therapy and correlation with migration patterns. (A) Comparison of CD15 and CD16 expression of aCML neutrophils after 2 weeks of ruxolitinib treatment (2 weeks Ruxo) after two different purification methods: density gradient centrifugation (density gradient, left) and negative magnetic isolation via MACSxpress® separation (MACS, right). (B) Changes in CD16, CD15 and fMLPR (top) as well as CXCR1 and CXCR2 (bottom) expression given as mean fluorescent intensity (mfi) of aCML neutrophils over the course of therapy. Black triangles and black solid lines indicate the aCML patient (every timepoint n = 1), while grey dots and grey dashed lines indicate the median and the grey dotted lines indicate the interquartile range of the age-matched controls (n = 5). Numbers label the specific values for receptor expression at the respective timepoint. (C-F) Correlation of receptor expression against migration upon corresponding stimulus treatment. Black triangles indicate the aCML patient (n = 1) and grey dots indicate age-matched controls (n = 5). Correlations were computed using GraphPad Prism™. Spearman r and p-value for the correlation of aCML samples are given. (C) fMLPR expression correlated against migration upon fMLPR treatment. (D) CXCR1 expression correlated against migration upon CXCL8 treatment. (E) CXCR2 expression correlated against CXCL1 treatment. (F) CXCR2 expression correlated against CXCL8 treatment. [file 12885_2020_7130_MOESM2_ESM.pdf]

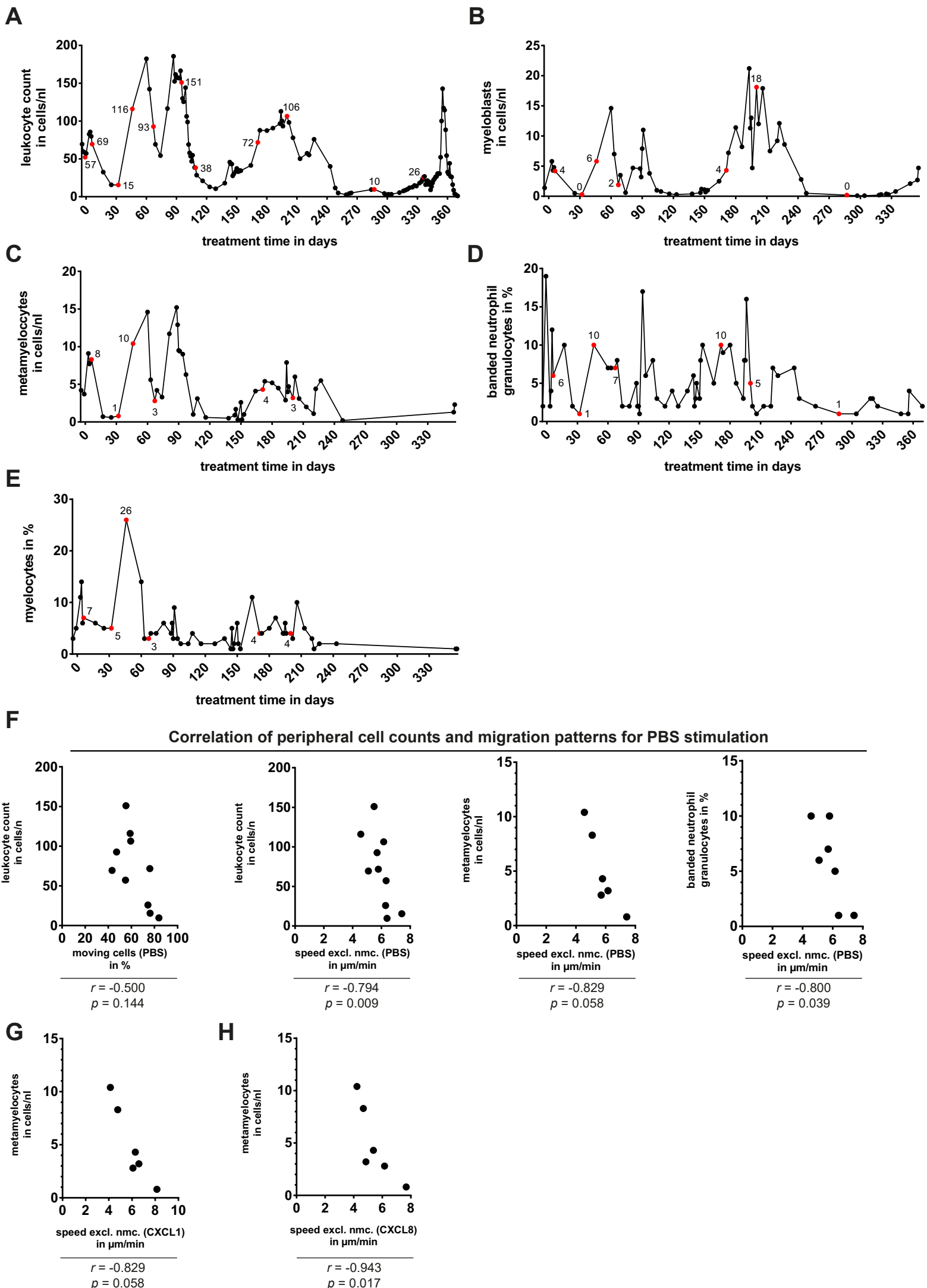

Supplement: Supplementary file 3 — Additional file 3: Figure S3. Changes in leukocyte parameters during ruxolitinib therapy and correlation of peripheral leukocyte counts with migration patterns. (A) – (E) Time course of chosen peripheral blood parameters of the aCML patient over the course of his disease and therapy. Displayed are the absolute WBC (A), myeloblast (B) and metamyelocyte (C) counts in cells/nl as well as the banded neutrophil granulocyte (D) and myelocyte (E) counts relative to the overall WBC in %. Red dots indicate timepoints where migration and flow cytometry data were acquired. (F) Correlation of peripheral cell counts with migration patterns upon PBS stimulation. From left to right: leukocyte counts with moving cells (PBS), leukocyte counts with speed excluding non-moving cells (PBS), metamyelocytes with speed excluding non-moving cells (PBS) and banded neutrophils with speed excluding non-moving cells (PBS). (G) Correlation of metamyelocyte counts with speed excluding non-moving cells upon CXCL1 treatment. (H) Correlation of metamyelocyte counts with speed excluding non-moving cells upon CXCL8 treatment. (F) - (H) Correlations were computed using GraphPad Prism™. Spearman r and p-values are given for each correlation below the graph. [file 12885_2020_7130_MOESM3_ESM.pdf]
